# The Kv7 channel activator, retigabine, induces vasorelaxation via an endothelial-independent pathway in male mouse aorta

**DOI:** 10.20463/jenb.2018.0024

**Published:** 2018-09-30

**Authors:** Hyun Namgoong, Chaeeun Cho, Sewon Lee

**Affiliations:** 1 Department of Human Movement Science, Incheon National University, Incheon Republic of Korea

**Keywords:** Kv7, retigabine, linopirdine, aorta, smooth muscle

## Abstract

**[Purpose]:**

Previous studies have indicated that Kv7 channels have an important role in the regulation of blood vessel reactivity, including in the coronary, renal, and cerebral arteries. The present studies examined whether Kv7 channels regulated vascular reactivity in the mouse aorta and investigated the mechanisms involved in the reactivity.

**[Methods]:**

Wild-type (WT) male C57BL/6 mice, between 10 and 15 weeks old, were used in this study. The vascular function of the aorta in WT male mice was assessed by using a pin myography system (Model 620; DMT, Denmark).

**[Results]:**

Vasorelaxation by an endothelial-dependent vasodilator, acetylcholine (ACh, 1 nM – 10 μM) and an endothelial-independent vasodilator, sodium nitroprusside (SNP, 1 nM – 10 μM) was induced in the aorta in a dose-dependent manner. Pre-incubation with the nitric oxide synthase inhibitor, L-NAME (100 μM, 20 min), completely abolished ACh-induced vasorelaxation, but did not block retigabine-induced vasorelaxation, which suggested that retigabine caused vasorelaxation in the aorta via smooth muscle activation rather than via endothelial cells. Pre-application of the Kv7 channel blocker, linopirdine (10 μM), resulted in a greater contractile response compared with that induced by vehicle in the aorta. In addition, pre-incubation with linopirdine (10 μM, 20 min) reduced retigabine-induced vasorelaxation (1–50 μM).

**[Conclusion]:**

This study has provided evidence that Kv7 channels may play a role in the regulation of aortic blood flow via smooth muscle activation.

## INTRODUCTION

Vascular smooth muscle cells express various types of potassium (K^+^) channels, such as voltage gated K^+^ (Kv), large conductance Ca^2+^-activated K^+^ (BKCa), ATP-sensitive K^+^ (ATP), inward rectifier K^+^ (Kir), and two-pore domain K^+^ (K_2p_) channels^1-3^. Of these K^+^ channels, Kv channels are widely expressed in the vasculature and are reported to modulate resting membrane potential (*E*m) and vascular tone in various types of blood vessels^4,5^. Because Kv channels are very important in the regulation of vascular tone, the alteration of these channels may be associated with pathophysiological conditions, such as hypertension^6-8^ and diabetes^9-11^. Among Kv channels, those encoded by *KCNQ*1–5 (Kv7.1–7.5) are a family of voltage-dependent ion channels that have also been shown to regulate resting Em and vascular contractility in various blood vessels^12-15^. Kv7 channels are expressed in the vasculature of a number of species, such as mice^16,17^, rats^14,18,19^, pigs^12,15^, and humans^13,20^. Previous studies have indicated that Kv7 channels play an important role in the regulation of blood vessel tone, including in the coronary, renal, and cerebral arteries^14,18,21^. In addition, Kv7 channels have been identified as effective regulators of vascular smooth muscle contractility in various blood vessels from normotensive animals^22^. However, there is limited information about the function of these channels in the mouse aorta. Thus, the present studies examined whether Kv7 channels regulated vascular reactivity in the mouse aorta and which mechanisms, endothelium-dependent or-independent, were involved in the reactivity.

## METHODS

### Chemicals

General chemicals and reagents, including acetylcholine (ACh), sodium nitroprusside (SNP), *N*^G^-nitro-l-arginine methyl ester (L-NAME), phenylephrine (PE), retigabine, linopirdine, and ethyl alcohol, were purchased from Merck (Gyeonggi-Do, South Korea). Dimethyl sulfoxide (DMSO) was purchased from Mentos Biotechnology (Gyeonggi-Do, South Korea). Retigabine (50 mM stock) was made in DMSO, whereas linopirdine (10 mM stock) was dissolved in ethyl alcohol. Vehicle control experiments were also performed.

### Animal experiments

All experimental protocols were approved by the Animal Care and Use Committee of Incheon National University (INU 2017-11). Wild-type C57 BL/6 mice, between 10 and 15 weeks old, were used in this study. The mice were housed in a temperature-, humidity-, and light-controlled (12 hour light/dark cycle) animal facility and given free access to standard mouse chow and water. All mice were anesthetized by an intraperitoneal injection of 2.5% tribromoethanol (0.01 mL/g of body weight).

### Isometric tension recordings

Under anesthesia, thoracic aortas were cut into 2 mm rings, isometrically mounted into a pin myography system (Model 620; Danish Myo Technology, Aarhus, Denmark), and maintained in physiological saline solution (PSS) in an atmosphere of 95% O_2_ and 5% CO_2_ at 37°C for the entire myography experiment. Continuous recordings of changes in the tension were acquired by using a PowerLab system (AD instruments, Colorado Springs, CO, USA). After a 30 min equilibration period, the aortas were placed under a pretension of 15 mN and equilibrated for a further 30 min. All aortas were stimulated with the cumulative addition of high K^+^ (50 and 100 mM KCl) to check the viability of the aortic rings. Based on previous studies^23,24^, the aortic rings were pre-contracted with 1 μM PE for 15 min to achieve a stable level of basal constriction to determine the subsequent vasorelaxation response. After stable constriction was induced, the concentration-response curves were acquired by the cumulative addition of the Kv7 activator, retigabine (1–50 μM). The degree of vasorelaxation at each concentration was measured and expressed as the percentage of the force generated in response to PE. In another experiment, linopirdine (10 μM) or an inhibitor of nitric oxide (NO) synthase, L-NAME (100 μM), was pre-incubated for 20 min to verify that they blocked Kv7 channel activation or nitric oxide production. Subsequently, retigabine (1–50 μM) was used in the pre-incubated blood vessels to confirm whether the vasodilation response occurred. In other experiments, after pre-contraction of the vessels with 1 μM PE, 1 nM–10 μM ACh or 1 nM–10 μM SNP-induced vasorelaxation was assessed to examine the vascular response.

### Statistical analysis

All data were presented as the mean ± standard error of the mean. To compare the means of two groups, the unpaired t-test was used. Two-way ANOVA followed by Bonferroni’s post-hoc test was performed to determine the significance between groups. All statistical analyses were computed by using GraphPad Prism (version 6.05, La Jolla, CA, USA). The significance of differences was accepted at P-values of <0.05, unless otherwise stated.

## RESULTS

### Effects of Kv7 channel activators on acetylcholine and sodium nitroprusside-induced vasodilation

**Fig.1.**
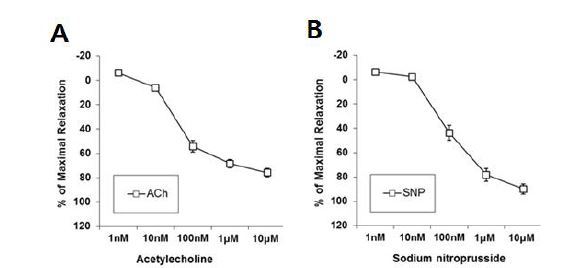
Vasorelaxation effects of acetylcholine (ACh) and sodium nitroprusside (SNP). A, Vasorelaxation by the endothelial-dependent vasodilator, acetylcholine (ACh 1 nM – 10 μM, n=28 rings); and B, Endothelial-independent vasodilator, sodium nitroprusside (SNP 1 nM – 10 μM, n=11 rings).

We first examined whether an endothelial-dependent vasodilator, ACh, and an endothelial-independent vasodilator, SNP, induced vasorelaxation in the aorta of male mice. Vasorelaxation by ACh (1 nM – 10 μM) or SNP (1 nM – 10 μM) was induced in the aorta in a dose-dependent manner, suggesting that both the endothelial and smooth muscle cells in the aorta were not impaired by the dissection procedures (Fig. 1).

### Effect of retigabine after pre-incubation of L-NAME

**Fig.2.**
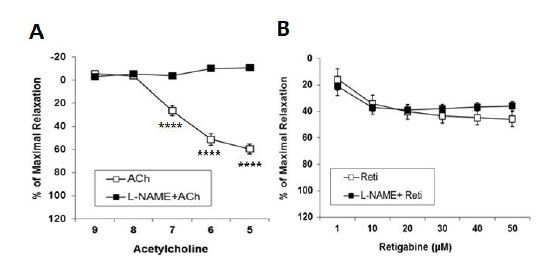
Effect of retigabine in the aorta from male mouse. A, Effect of acetylcholine (ACh) after pre-incubation with L-NAME (100 μM 20 min, n=12). B, Effect of retigabine after pre-incubation with L-NAME (100 μM 20 min, n=10). ^****^P<0.0001 (Two-way, repeated measures ANOVA with Bonferroni multiple comparison post-hoc test).

The experiment was undertaken to assess whether the Kv7 channel activator, retigabine, induced vasorelaxation through the endothelial-derived relaxing molecule NO in aortic segments. Pre-incubation with an NO synthase inhibitor, L-NAME (100 μM, 20 min), completely abolished ACh-induced vasorelaxation, whereas pre-incubation with L-NAME did not block retigabine-induced vasorelaxation, which suggested that retigabine caused vasorelaxation via the activation of smooth muscle cells, rather than endothelial cells, in the aorta (Fig. 2).

### Effects of Kv7 channel inhibitor

**Fig.3.**
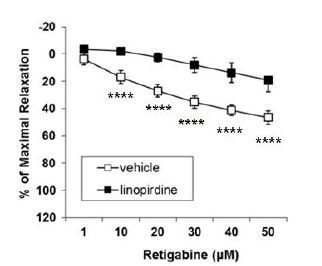
Effect of retigabine after pre-incubation with linopirdine (10 μM 20 min, n=11). ^****^P<0.0001 (Two-way, repeated measures ANOVA with Bonferroni multiple comparison post-hoc test).

We also tested whether a non-selective Kv7 channel inhibitor, linopirdine, abolished retigabine-induced vasorelaxation. Pre-incubation with linopirdine (10 μM, 20 min) reduced retigabine-induced vasorelaxation (1–50 μM) in the aorta, compared with vehicle-treated samples (Fig. 3).

### Effect of linopirdine on the contraction

**Fig. 4.**
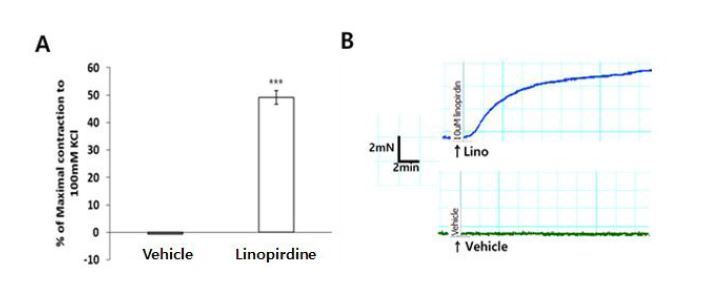
The percentage of contraction induced by linopirdine in comparison with the maximal contraction induced by 100 mM KCl (A). Representative traces showing responses to 10 μM linopirdine vs. vehicle (ethyl alcohol, B). ^***^P<0.001 (unpaired t-test).

Previous studies have suggested that the Kv7 channel blocker, linopirdine, exerts a depolarizing effect on vascular smooth muscle cells and causes constriction in the cerebral, renal, and coronary arteries^21,25,26^. Experiments were performed to test whether the Kv7 channel was involved at rest condition in the mouse aortas. Pre-application of the Kv7 channel blocker, linopirdine (10 μM), resulted in a greater contractile response in mouse aorta compared with vehicle (Fig. 4).

## DISCUSSION

Vascular smooth muscle Kv7 channels are known as modulators of vascular and vasodilatory responses^14,21,25^. Previously, we have shown heterogeneity between vascular beds, with an emphasis on the difference in protein expression of Kv7 channels in the cerebral and coronary arteries^25^. However, little is known about the role of Kv7 channels in macrovasculature such as the thoracic aorta. Specifically, it is unclear whether the activation of Kv7 channels may induce vasorelaxation via endothelial-dependent or -independent pathways in the aorta. Thus, the present study examined whether Kv7 channels regulated vascular reactivity and which mechanisms were involved in the regulation in mouse aorta. We have shown that ACh and SNP induce vasorelaxation in the aorta in the pre-contracted state induced by PE, which suggested that both the endothelial and smooth muscle cells of the aorta were not damaged during the incision process. Consistent with previous studies^23,24^, we confirmed that ACh and SNP induced vasorelaxation in the aortas. Next, we examined the effect of retigabine in the aorta of male mice. Pre-incubation with L-NAME completely abolished ACh-induced vascular endothelial function, but did not block retigabine-induced vasodilation. These results suggested that retigabine was an activator of Kv7 channels and caused vasodilation through direct action on vascular smooth muscle cells in the aorta. In addition, retigabine-induced vasodilation was attenuated by pre-incubation with linopirdine, which suggested that Kv7 channels may play an important role in the regulation of vascular reactivity in the aorta.

Previous evidence has shown that the nonselective Kv7 channel inhibitor, linopirdine, has a depolarizing effect on vascular smooth muscle cells, which induces vasoconstriction^21,22,26,27^. Specifically, the application of linopirdine induced 10–12% of the contraction induced by 100 mM KCl in the cerebral artery of mice^25^. In the present study, the application of linopirdine induced over 40% constriction compared with 100 mM KCl in the aorta. Linopirdine is an inhibitor of the Kv7 channels and may be assumed to affect a relatively larger portion of Kv7 channels in the aorta than in the cerebral artery. These results suggested that Kv7 may play an important role in the regulation of vascular reactivity in macrovessels, such as the thoracic aorta, compared with the microvasculature. Kv7 channels may be expected to have certain effects on vascular reactivity through various pathways in vascular diseases, such as chronic hypertension, diabetes, hyperlipidemia, and atherosclerosis, which can cause vascular dysfunction. Therefore, further study of Kv7 channels may provide pharmacologically or pathologically important basic data for vascular disease.

In summary, the results of this study demonstrated that Kv7 channels were able to mediate vasorelaxation in the aorta and that this reactivity occurred through vascular smooth muscle cell activation rather than endothelial cells.
